# RETRACTED ARTICLE: Periacetabular osteotomy: an analysis of social media to determine the most common questions asked by the periacetabular osteotomy population

**DOI:** 10.1186/s12891-024-07249-9

**Published:** 2024-02-17

**Authors:** John M. Gaddis, Bretton Laboret, Ryan Bialaszewski, Elizabeth Bergman, Jenny LaCross, Edward Mulligan, Joel Wells

**Affiliations:** 1 University of Texas Rio Grande Valley School of Medicine, 1201 West University Drive, Edinburg, TX 78501 USA; 2 University of Texas Southwestern Medical School, Dallas, TX USA; 3 Texas Woman’s University, Denton, TX USA; 4 Tufts University Doctor of Physical Therapy Program – Phoenix, Phoenix, AZ USA; 5 Baylor Scott and White Hip Preservation Center, Department of Orthopedic Surgery, McKinney, TX USA

The Editor has retracted and removed this article. After publication, concerns were raised regarding the ethical component of the study, specifically access to private groups and use of identifiable patient data from social media without ethics approval or consent. Fig. 1 contains visible profile pictures from private social media groups, further increasing the risk of participant identification. The authors were unable to provide evidence of consent from the members of the private Facebook groups where part of the data was procured. Therefore, the Editor has lost confidence in the integrity of this article. This article has been removed from the online version of BMC Musculoskeletal Disorders.

Authors Edward Mulligan, Elizabeth Bergman, and Jenny LaCross agree with this retraction. The other authors did not respond to correspondence from the Publisher about this retraction.

